# Oncolytic Viruses as Anticancer Vaccines

**DOI:** 10.3389/fonc.2014.00188

**Published:** 2014-07-21

**Authors:** Norman Woller, Engin Gürlevik, Cristina-Ileana Ureche, Anja Schumacher, Florian Kühnel

**Affiliations:** ^1^Clinic for Gastroenterology, Hepatology and Endocrinology, Medical School Hannover, Hannover, Germany

**Keywords:** oncolytic virotherapy, oncolytic virus, antitumor immunity, antitumor immune response, oncolytic agents

## Abstract

Oncolytic virotherapy has shown impressive results in preclinical studies and first promising therapeutic outcomes in clinical trials as well. Since viruses are known for a long time as excellent vaccination agents, oncolytic viruses are now designed as novel anticancer agents combining the aspect of lysis-dependent cytoreductive activity with concomitant induction of antitumoral immune responses. Antitumoral immune activation by oncolytic virus infection of tumor tissue comprises both, immediate effects of innate immunity and also adaptive responses for long lasting antitumoral activity, which is regarded as the most prominent challenge in clinical oncology. To date, the complex effects of a viral tumor infection on the tumor microenvironment and the consequences for the tumor-infiltrating immune cell compartment are poorly understood. However, there is more and more evidence that a tumor infection by an oncolytic virus opens up a number of options for further immunomodulating interventions such as systemic chemotherapy, generic immunostimulating strategies, dendritic cell-based vaccines, and antigenic libraries to further support clinical efficacy of oncolytic virotherapy.

## Introduction

Oncolytic viruses are novel antitumor agents with the ability to selectively replicate and lyse tumor cells while sparing healthy tissue. This intriguing characteristic is either an inherent feature of certain virus species or a result of targeted genetic engineering, which harnesses tumor-specific molecular alterations for virus replication and tumor cell lysis (1). The ideal and intriguing concept has been that the oncolytic virus infection proceeds throughout the whole tumor, thereby leading to effective tumor cell lysis until the rim of malignant tissue is being reached and further infection is kept in check. Although numerous oncolytic viruses have been generated according to this concept, first clinical trials did not meet the high expectations that have been raised by promising preclinical developments (2). Though clinical benefit by these first wave oncolytic agents, such as the mutated Adenovirus (Ad) Onyx-015 has been rather modest, these studies confirmed that oncolytic viruses can be safely administered in human patients and may also work synergistically with systemic radio- or chemotherapy (3). H101, a direct derivative of the E1B55k-deleted Onyx-015, was approved in China in 2006 being the first clinically applicable oncolytic virus (4). At the same time, many factors have been recognized, which severely impair therapeutic efficacy of oncolytic viruses such as virus neutralization by blood components, ineffective transduction of tumor tissue, intratumoral stromal barriers that inhibit virus spread, hypoxic conditions, interstitial pressure, and finally, the rapid immune-mediated elimination of the virus from the tumor tissue (5).

Apart from the cytoreductive aspect, oncolytic viruses have been initially developed for, it has become increasingly clear during the recent years that virotherapy exerts multiple antitumoral activities. These include direct effects by cytotoxic cytokines released upon infection by tumor-resident or infiltrating immune cells (6, 7). Also, effects on the tumor vasculature have been demonstrated (8, 9). In contrast to the notion that the host’s immune system limits the efficacy of virotherapy by rapid clearance of infection, it has been perceived that collateral induction of innate and adaptive immune responses against the tumor essentially contributes to therapeutic efficacy of virotherapy (10). Oncolytic virus-mediated destruction of tumor tissue activates innate immune receptors once the immunogenic cell debris is taken up and cross-presented by antigen-presenting cells. Antigen-presenting cells are additionally activated by signals coming from innate cells and the damaged tissue. The local inflammation of tumor tissue during oncolytic virus infection therefore provides suitable conditions for the triggering of tumor-directed immune responses (11, 12). Oncolytic viruses that are currently most advanced in clinical development have been designed to amplify the *in situ* vaccinative and immunostimulatory effect of virus infection. The GM-CSF-expressing oncolytic vaccinia virus JX-594 has shown promising results in phase I/II clinical studies in hepatocellular carcinoma (13). In advanced melanoma, the GM-CSF-expressing herpes virus T-Vec led to a significant number of durable responses and improved survival in a phase III trial in human patients, thus demonstrating clinical efficacy of virotherapy in human cancer patients (14).

There has been evidence that virotherapy may profit from general immunosuppression by increased intratumoral virus spread and by delayed virus clearance (15). Apart from safety aspects, the increased immediate tumor response due to oncolysis would be in this case achieved at the cost of losing effective tumor-antigen cross priming and the perspective of long-term antitumoral efficacy. In this review, we want to deliver a closer look on how oncolytic viruses induce and shape tumor-antigen directed immune responses. First, we want to address the origin of antitumoral immune responses on the level of the infected tumor cell by discussing the role of viral oncolysis for induction of immunogenic cell death (ICD). The aspect of ICD has also been recently reviewed in depth by Bartlett et al. providing complementary information on how armed viruses and combination strategies work to enhance antitumor immunity (16). In the second part of our review, we want to shed light on the role of several immune cells populations that contribute to the tumor microenvironment. Finally, we want to highlight some current trends and developments exploiting the immunostimulatory and vaccinative potential of oncolytic virotherapy to raise T cell responses against the tumor mutanome.

## Oncolytic Virus-Mediated Cell Death Mechanisms

Viruses, mainly DNA viruses, need time after cell entry to complete the viral life cycle and have consequently developed elaborate strategies to hide from being detected by the host’s immune system (17). The requirement of effective “stealth” mechanisms illustrates that virus-mediated cell killing can be a highly immunogenic way for cells to die. This perception has been exploited in vaccinations for a long time since vaccines can be more potent when delivered and expressed by viral vectors (18). Due to the fundamental relevance in multiple physiological processes, enormous efforts have been made to understand the immunological consequences of different kinds of cell death, which have been classified into three major kinds: apoptosis, necrosis, and autophagy (19). Apoptosis is mainly characterized by defined morphological changes such as formation of apoptotic bodies and biochemical signaling such as caspase activation and loss of mitochondrial membrane integrity. Flipping of phosphatidylserines to the outer membrane surface during apoptosis facilitates silent removal of apoptotic bodies by phagocytes. This process is usually accompanied by release of anti-inflammatory cytokines to minimize immune-mediated collateral damage (20). The coordinated cell demise by apoptosis is essential for normal development and tissue homeostasis and has been therefore regarded for long time as a non-immunogenic or even a tolerogenic event. A second cell death type, necrosis, appears to be a less coordinated process and the biochemical pathways have been much less intensively studied. Necrosis is characterized by swelling of organelles and cytoplasm followed by rupture of the plasma membrane with release of cytoplasmic contents. Since necrosis is frequently accompanied by release of proinflammatory cytokines such as tumor necrosis factor-α (TNF-α) (21), and other immune activating mediators, necrosis has been more or less regarded as being immunogenic. However, the traditional perspective of non-immunogenic/tolerogenic apoptosis and immunogenic necrosis has been challenged by the finding of “immunogenic” apoptosis in tumor cells, which can be induced by specific chemotherapies such as anthracyclines and oxaliplatin (22, 23). When mice were treated with tumor cells that have been killed by these “ICD” inducers, long-term immunity against a challenge with the same tumor could be observed whereas other chemotherapeutic agents failed to induce antitumoral immunity. Since then, several other systemically applicable ICD inducers have been described (24).

Oncolytic virus-mediated cell death does not exactly follow the classical schemes of apoptosis or necrosis but rather displays specific features of both cell death modalities with some variation between different oncolytic virus types. Accordingly, terms like programed apoptosis, necroptosis, pyroptosis, or necrosis-like programed cell death have been used to describe cell death by different oncolytic virus species, trying to describe the coordinated manner in which cells are rearranged in the course of the viral infection cycle, and the membrane disruptive and inflammatory release of viral progeny and cytoplasmic/nucleic contents during lysis. Necrosis-like programed cell death has been observed using oncolytic Ads (25). Though activity of caspases could be observed, p53 activity and mitochondrial pathways were effectively blocked whereby execution of cell death was essentially independent of caspase activation. Likewise, programed necrosis was also observed in cells infected with an oncolytic vaccinia virus. Though some limited features of apoptosis and autophagy were detectable such as phosphatidylserine exposure and LC3 lipidation, necrotic morphology predominated and the necrotic process was also identified as causative cell death modality (26).

Recently, receptor-interacting protein kinases RIP1 and RIP3 have emerged as a decisive switch from immunologically silent apoptosis to necrotic inflammation (27). Once caspase-8 activity, located in a receptor-associated complex called necrosome, is suppressed, e.g., by a pathogen-encoded inhibitor, RIP1 is stabilized, then attracting and phosphorylating RIP3 (28). RIP3 activation phosphorylates the major downstream target mixed lineage kinase domain-like (MLKL) by phosporylation and trimerization that translocates to the plasma membrane to mediate Ca^2+^ influx and initializing membrane rupture (29). RIP1/RIP3-dependent necroptosis therefore appears to function like a backup mechanism allowing the elimination of pathogen-infected cells that cannot undergo apoptosis (30). Necrotic features of RIP3-dependent cell death are necessary for induction of inflammation, improved antigen presentation and effective defense against the pathogen. It has been demonstrated that the highly specific caspase-8 inhibitor vICA, encoded by cytomegalovirus, predisposes to RIP3-dependent necrosis (31). Additionally, CrmA related apoptosis inhibitors activate TNFR-dependent necroptosis in vaccinia virus infections in mice augmenting clearance of the virus (32). Interestingly, cytomegalovirus also express a RIP3 inhibitor, vIRA, which blocks this “backup” cell death pathway to reduce inflammatory responses (33). A downstream target of the RIP1-RIP3-necrosome in necroptosis is JNK-1 and its substrate c-Jun with a final impact on the production of reactive oxygen species (ROS) (34). We could show that oncolytic Ad infection in human tumor cells strongly induced JNK-1 activation, downstream phosphorylation of c-Jun, and activation of other stress-activated kinases (35). It has further been shown that programed necrosis by oncolytic vaccinia virus infection involved formation of a RIP1/Caspase-8 complex (26). In this study, the relevance of RIP1 in vaccinia virus-induced programed necrosis was demonstrated by pharmacological inhibition of both RIP1 and downstream targets including MLKL, which significantly attenuated necrotic cell death. Using an oncolytic influenza viruses, armed with the antitumoral cytokine IL-24, it has been shown that IL-24 turned cell death, mediated by a TLR3-associated, RIP-1 containing signaling complex, into a pure apoptotic phenotype by unleashing caspase-8 activity (36). Though enhanced tumor cell killing was observed *in vitro*, the consequences of this approach on immunogenicity and antitumoral immune responses *in vivo* are unclear.

In summary, the RIP1/RIP3 necrosome plays a central role in induction of inflammation and virus-mediated ICD and is therefore an interesting target for more detailed investigations, and targeted modulation in oncolytic virotherapy. Again, it has to be considered that enhanced immunogenicity of oncolytic virus-mediated cell death will probably affect viral spread.

## The Role of Autophagy in Oncolytic Virus-Mediated ICD

**Figure 1:**
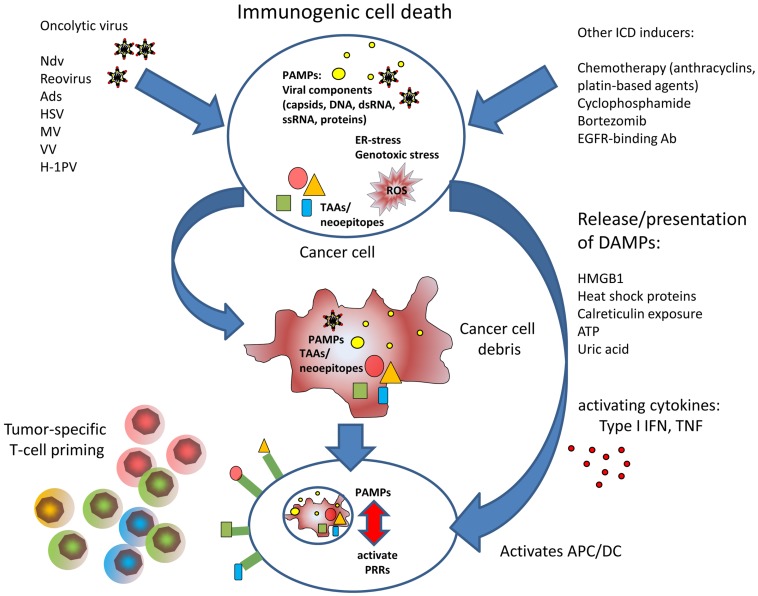
**The figure illustrates the improved T cell priming in oncolytic virotherapy**. Viral oncolysis of tumor cell induces immunogenic cell death by accumulation of PAMPs and accompanied by release of DAMPs. PAMPs and DAMPs activate antigen-presenting dendritic cells that can induce cytotoxic T cell responses against tumor-associated antigens or neoepitopes, respectively.

Another cell death type, autophagy, is a process that leads to self-digestion of organelles after inclusion in cytosolic lysosomes (autophagolysosomes). Since signs of autophagy also occur as a reversible process in the context of nutrient starvation, it is not completely clear whether autophagy is causative for cell death or is an epiphenomenon of other cell death triggers. However, autophagy plays a definitive role in triggering immune responses. Autophagic mechanisms are involved in the clearance of intracellular microbial or viral pathogens not only by intracellular digestion but also by improved processing of microbial/viral antigens for antigen presentation on MHC I as known for herpes simplex virus infections (37). Autophagy can be a part of a cellular reaction to infection by oncolytic viruses, which has been observed first in glioma treatment with oncolytic Ads (38, 39). Induction of autophagy has also been demonstrated for Newcastle disease virus (NDV) (40). In both cases, investigations using the autophagy inducer rapamycin suggested that autophagy augments viral replication and propagation and may lead to improved antitumor responses (41, 42). An interesting subtype of autophagy, called mitophagy, has been reported recently (43). The authors have shown that attenuated measles viruses of the Edmonton strain exploit selective reduction of mitochondria via SQSTM1/p62-mediated mitophagy for enhanced viral replication. Mitophagy resulted in decreased mitochondrion-bound mitochondrial antiviral signaling protein (MAVS) thus weakening the innate immune response mediated by RIG-I-like receptors. In summary, cell death by oncolytic viruses displays signs of apoptosis, autophagy, and necrosis to a variable extent. What all oncolytic viruses have in common is the immunogenic nature of virus-induced cell death (see also Figure 1 for an overview). The determinants characterizing ICD are summarized in the next chapter.

## Inducers and Mediators of Immunogenic Cell Death: DAMPs and PAMPs

Antigen-presenting cells such as dendritic cells (DC) fulfill a central role in triggering effective T cell responses in case of a pathogenic threat. Antigen-presenting cells are activated when encountering pathogen-derived structures, called PAMPs (pathogen-associated molecular patterns), which reflect conserved components of microbes and viruses. Classical PAMPs are microbial DNA with unmethylated CpG, defective viral genomes that occur during viral lysis, double stranded RNA, single stranded RNA, 5′-triphospate RNA, lipoproteins, surface glycoproteins, and bacterial membrane components such as LPS. PAMPs are recognized by pattern recognition receptors (PRRs) present on innate immune cells, antigen-presenting cells, and also on epithelial cells. PRR include toll-like receptors, retinoid acid inducible gene I (RIG-I)-like receptors (RLRs), AIM like receptors (ALRs), and nucleotide-binding oligomerization domain (NOD)-like receptors (NLRs) (44). In 1994, the “danger” hypothesis by Polly Matzinger (45) brought up the idea that, besides the classical feature to distinguish between self and non-self, the immune system must be able to adequately respond to tissue distress, and that this additional competence requires molecular signaling coming from affected tissue. According to this hypothesis, molecular danger signaling immediately alerts innate immune cells and facilitates their attraction to the site where ICD occurred. Furthermore, danger signaling must activate DCs to provide for the stimulation needed to activate antigen-specific T cells. A number of molecular factors called danger-associated molecular patterns or DAMPs have been described functioning as such danger signals to orchestrate attraction of innate immune cells, phagocytosis of immunogenic cell debris, and to activate effective T cell priming. Some DAMPs are immune activating cytokines such as TNFs or type I interferons that can be immediately emitted in response to threat. Other factors are metabolites that create a chemotactic gradient for innate immune cells corresponding to a “find me” signal. Further, DAMPs already reflect signs of structural damage caused by the infection process. Externalized proteins, more or less linked to the membrane of the infected cell can provide an “eat me” signal to attracted phagocytes. When cells undergo immunogenic apoptosis, the release of ATP is a known “find me” signal to promote phagocytic clearance of those cells at a very early time point (46, 47). ATP is released by Pannexin channels and sensed by P2Y (2) purinergic receptors on monocytes to facilitate their attraction to the site of apoptotic cell death. Additionally, ATP acts on P2X (7) purinergic receptors on DCs, thus activating the NLRP3 inflammasome (48). ATP has also been described being released by cells infected by oncolytic viruses (49, 50). In induction of ICD, ATP can also act synergistically with another DAMP, cell surface exposed calreticulin or ecto-CRT (51). Calreticulin is under physiological conditions located in the lumen of the endoplasmic reticulum (ER). However, dying cells externalize and present calreticulin on their surface where it serves as a potent “eat me” signal to phagocytes (52). It has been shown that calreticulin is exposed on the cell surface of lung adenocarcinoma cells after treatment with an oncolytic coxsackievirus B3 (50). Ecto-CRT has also been observed with several oncolytic Ads (49, 53).

When cells succumb to necrosis, they also externalize and release the high mobility group box 1 (HMGB1) protein into the cellular environment, which is known for its proinflammatory properties (54). The relevance of HMGB1, Ecto-CRT, and ATP in characterizing ICD has facilitated reliable high throughput screens for ICD-inducing agents (55). HMGB1 release has been observed with multiple oncolytic viruses, e.g., Ad, Vv, and Mv (26, 53, 56, 57).

Further, important DAMPs are released heat shock proteins, such as HSP70 and HSP90, and uric acid. Heat shock protein release has been demonstrated to play a role in induction of tumor-specific immune responses by the oncolytic parvovirus H1 (58). Uric acid is a product of nucleic acid catabolism and constitutively present in the cytosol of normal cells in high concentrations that can even rise in stress situations due to increased DNA/RNA degradation. Even the debris of dead cells is able to continue production and release of uric acid providing a sustained danger signal (59). It is believed that a chemical phase change to urate microcrystals at supersaturated loci is the actual immune activating event. Using the oncolytic Ad Telomelysin, it has been shown that infected tumor cells produced uric acid, which in turn stimulated IFN-γ and IL-12 secretion by DC and supported the induction of cytotoxic T cells (60). The DAMPs described so far represent potent immune activators in case of immunogenic apoptosis or necrosis. However, also cell-intrinsic inhibitors of DAMPs exist. Recent results showed that the cellular peptidases dipeptidylpeptidase 3 (DPP-3) and thimet oligopeptidase 1 (TOP-1) present in and released by necrotic cells were able to provide a non-immunogenic signal and inhib antigen cross presentation (61). Since inhibition of the peptidases restored immunogenicity and antigen-specific Tcell priming, interfering with these mechanisms in oncolytic virus-mediated cell death could be a promising option to enhance immunogenicity.

## The Role of ER-Stress in Oncolytic Virus-Mediated ICD

A further important mechanism that provides dying cells with an immunogenic signature is ER-stress. The ER is a central production site for proteins and membrane components involved in the secretory pathway. The ER is also an important sensor for ER-stress, a physiological reaction to dysbalanced protein synthesis, e.g., in the context of viral infections. Under homeostatic conditions, the luminal ER-stress sensors IRE1α, ATF6, and PERK are bound and silenced by the molecular chaperone Grp78/BiP. Once unfolded proteins accumulate in the ER due to an unphysiologic increase in protein synthesis, Grp78/BiP is competitively displaced from the ER-stress sensors leading to their subsequent activation for downstream induction of an unfolded protein response (UPR) (55). Whereas activation of IRE1α and ATF6 leads to expression of compensatory acting genes, PERK/ATF activation facilitates phosphorylation of eIF2α to induce a general stop of translation until ER-stress has been released. eIF2α-dependent shutdown of translation is also an intrinsic defense reaction to prevent that intracellular pathogens from occupying the protein synthesis machinery for their own purposes. Consistent with this function, ER-stress can confer a significant immunogenic signal to dying cells, which has been demonstrated using chemotherapeutics that are able to directly induce ER-stress (55). According to the relevance of ER-stress as pathogen sensor, many viruses have evolved elaborate ways to circumvent or to adopt ER-stress pathways to their benefit and interfere with ER-stress pathways and UPR (17). ER-stress pathways are also an interesting target to modulate the outcome of oncolytic virutherapy and to increase ICD. Genome-wide RNAi-screens for host factors that modulate viral oncolysis showed that ER-stress and UPR are highly important modulators of viral oncolysis by rhabdovirus (62). To confirm the screening results, the authors showed that inhibition of IRE1α dramatically improved rhabdovirus-mediated oncolysis. Accordingly, ER-stress has been a promising mechanism for pharmacological interference to support viral oncolysis. Bortezomib is a clinically approved inhibitor of the 26S proteasome and leads to collateral ER-stress and ICD with both apoptotic and necrotic features. We showed that low-dose bortezomib enhanced immunogenic tumor cell killing and antitumoral T cell responses in hepatocellular carcinoma models in mice (35). Another study showed that Reovirus and bortezomib synergistically induced apoptosis in multiple myeloma (63). In case of oncolytic herpes simplex virus (oHSV), it could be recently demonstrated that bortezomib-induced UPR even increased virus replication thus leading to enhanced, synergistic tumor effects (64).

## Oncolytic Virus Infection Disrupts the Tumor Microenvironment

Immunogenic cell death is basically the first aspect in innate and adaptive immune effects that have been recognized as a central mode of action in virotherapy (65). The tumor microenvironment also essentially contributes to the triggering of antitumoral immunity. Tumors not only consist of tumor cells but also of stromal fibroblasts, endothelial cells and resistant leukocytes which together with the extracellular matrix constitute the tumor microenvironment. Intratumoral infection by an oncolytic virus is not only a dramatic impact for tumor cells but is also disruptive for tissue architecture and immune homeostasis within the tumor microenvironment. The effect of the tumor stroma to oncolysis is a most enigmatic and barely understood phenomenon since fibroblasts are relatively resistant to virus infection and generate important intratumoral barriers that inhibit virus distribution. To address these barriers, it has been tried to interfere with stroma integrity by oncolytic viruses expressing collagenase and matrix-modifying enzymes (66, 67). The activation of the innate immune system following intratumoral virus infection represents the first defense wave of the host reaction to tumor lysis. Tumor-resident innate immune cells become modulated by inflammatory cytokines that are immediately released upon contact of macrophages with viral structures (68, 69). Further innate immune cells invade the damaged tumor tissue and induce an acute inflammation to fight the viral infection. Neutrophils invade the oncolytic tumor and contribute to immediate antitumoral cytotoxic effects (9, 70). Additional neutrophil-activating signals have been used to increase this effect of oncolytic virotherapy (71). Interestingly, in case of measles virus, it has been shown that attenuated, oncolytic viruses can be even better neutrophil activators compared to their wild-type counterparts (72). Results of several studies suggested that the innate immune response should be suppressed to enhance oncolytic virus propagation and intratumoral spread (73–76). It has also been shown with measles virus that innate immune cytokines can confer resistance to tumor cells against virus-mediated lysis (77). However, the innate immune response is an essential interface for triggering of adaptive immune response including long-term antitumoral T cell responses. It could be rather promising to selectively address suppressive innate immune cell subpopulations in oncolytic virotherapy (6). Since the oncolysis-mediated modulation of the tumor microenvironment decisively governs the priming of adaptive immune responses, the individual immune cell types that contribute to the tumor microenvironment and the immediate reaction to viral oncolysis need a more detailed description.

## Myeloid Cells

Aside of neutrophils, macrophages and monocytes belong to the initial defense response by the innate immunity against pathogens. These populations are highly activated after viral infections, are capable of phagocytosis, support the professional antigen-presenting cells, and contribute to adaptive immunity. Within an intact tumor, secretion of immunosuppressive cytokines determines the phenotypic differentiation of these innate immune cells to adopt an immunosuppressive status to promote tumor progression and metastases (78). Consequently, the immunosuppressive phenotype of these cells can interfere with therapeutic antitumor immune activities. Macrophages residing in the tumor microenvironment have been designated as tumor-associated macrophages (TAMs) and can be divided into two groups, one showing an inflammatory M1 phenotype and the other showing, an immune suppressive M2 phenotype, the latter being overrepresented within the tumor microenvironment (79). It is known that viral inflammation can polarize macrophages toward an M1 phenotype (80). This population promotes inflammatory conditions and supports the triggering of antigen-specific immune response. It has been shown that TAM depletion by chlodronate liposomes prevent intratumoral virus clearance resulting in increased replication and virus spread resulting in improved antitumoral effects (81). Like macrophages, tumor-associated neutrophils can be either assigned to an inflammatory N1 phenotype or an immune suppressive N2 phenotype, respectively (82). Though invading neutrophils belong to the first infiltrating immune populations at the site of inflammation (9), the role of neutrophil polarization in oncolytic virotherapies has not yet been addressed.

In recent years, myeloid-derived suppressor cells (MDSC) population has been described as one of the most important immunosuppressive within the tumor microenvironment. These cells have been observed in primary tumors as well as in metastases of patients (83, 84). Myeloid suppressor cells are attractive targets for therapeutic investigations (85). Related to oncolytic virotherapy, it was shown that the combination with gemcitabine, which is a chemotherapeutic agent depleting MDSC populations, increases antitumoral immune responses (86, 87).

## Virotherapy is a Potent NK Cell Activator

Among the cells of the innate immune system, NK cells play a crucial role in clearing viral infection and in fighting malignant cells. Trying to escape from adaptive immune responses by downregulation of MHC, virus-infected cells, and tumor cells become a natural target of NK cells. In line with a role of NK cells in immunoediting of tumors, tumor-infiltrating NK cells correlate with a favorable prognosis in humans (88). NK cells belong to the first immune cell populations that are activated by a virus-mediated inflammation in order to identify and directly kill virus-infected cells (89). This suggests that NK cell inhibition will significantly support intratumoral spread of oncolytic viruses and effective tumor lysis. A study using oncolytic VSV showed that the replication of the virus could be enhanced by NK cell depletion resulting in more effective tumor killing (74). The supportive effect of NK cell inhibition was confirmed by the same group by application of a virus encoding for UL141, which blocks CD155 on infected cells thereby interfering with NK cell recruitment and activation (90). Furthermore, it was shown that the NK cell natural cytotoxicity receptors (NCR) NKp30 and NKp46 were highly activated during oHSV resulting in effective killing of oHSV infected cells thus impeding viral spread and oncolytic therapy (75).

On the other hand, several studies showed an antitumoral effect of NK cells after oncolytic viral treatment. Depletion studies with VSV in the B16 melanoma model revealed an NK cell and T cell dependent tumor regression (91). Furthermore, the remodeling of the immunosuppressive tumor microenvironment of prostate cancer by the infection with oncolytic reovirus demonstrated a strong NK cell involvement in antitumoral immune response (92). It was also observed that the antitumoral effect of an oncolytic parapoxvirus ovis (ORFV) was mainly NK cell-mediated (93). Using an adenovirus expressing IFNβ for systemic NK cell activation, Suzuki et al. could show that intratumoral virus treatment in a pancreatic cancer model resulted in strong NK cell-mediated antitumoral cytotoxicity, when MDSC were eliminated by gemcitabine (86). These data illustrate that other immunosuppressive populations within tumor microenvironment play an important role in the establishment of antitumoral immunity, which must be considered for the role of NK cells in oncolytic virotherapy. Promising reports come from observations on the application as adjuvant to surgical tumor removal. This is of particular clinical relevance since surgery is still the most frequent therapeutic option with curative intention. In a first therapeutic approach using virotherapy as perioperative agent in a surgical stress model, Tai et al. showed that virotherapy by vaccinia virus or ORFV can release NK cell suppression during surgical intervention (94, 95). Virus-mediated NK cell activation effectively inhibited the engraftment of metastatic cells. This finding suggests that NK cells seem to be in particular efficient to protect against tumorigenic cells when an established immunosuppressive tumor microenvironment is lacking. These observations are supported by the increased antitumoral NK cell efficacy, when it is used with chemotherapeutic approaches like gemcitabine or cyclophosphamide, which are well known immunomodulatory agents with selective depletion effects on immunosuppressive populations like MDSCs or regulatory T cells (Treg), respectively (86, 96, 97). It was also demonstrated that a novel oncolytic rhabdovirus (Maraba MG1) was able to boost NK cell activity for the reduction of postoperative metastases (98). Intriguingly, the authors revealed that the effect of NK cell activation was mediated via virus infection of conventional DC. This interaction refers to the important function of DC as functional interface to innate immune effector cells for triggering adaptive immune responses. It is known from patients treated with cetuximab that NK cells are involved in antibody-dependent cytotoxicity of tumor cells and assist DCs in priming of antitumoral T cell responses by an NK:DC crosstalk (99). This aspect could be relevant in oncolytic virotherapy since antibody-mediated cell killing of tumor cells has already been shown to play a yet underestimated role in human patients who have been treated with an oncolytic vaccinia virus (100).

## Tregs and Treg Depletion during Oncolysis: Good or Bad?

Regulatory CD4 T cells (Tregs) are an immunosuppressive cell population that has frequently been discussed as a critical contributor to the tumor microenvironment. It has been shown that the ratio of intratumoral cytotoxic T cells and Tregs is a prognostic factor for the patient’s outcome and studies using antibodies blocking CTLA-4 (which is expressed on Tregs) for increased immune activation have shown that Tregs can be interesting targets for immunotherapeutic approaches (101, 102). The impact of viral infections on Tregs has been mostly studied in persistent or chronic virus infection, such as HCV or HBV whereas the role of Tregs during acute viral inflammations such as oncolytic virus infections is much less investigated. Studies showed that the number of Tregs significantly drops during acute viral inflammation to facilitate an effective antiviral immune response (103, 104).

To elicit enhanced immune stimulation, Treg depletion has therefore been considered a supportive measure during oncolytic virotherapy. Studies have shown that tumor preconditioning with IL-2 and Treg depletion using a depleting antibody or low-dose cyclophosphamide led to increased intratumoral uptake of systemically delivered reovirus or vesicular stomatitis virus. IL-2 in combination with Treg depletion generated “hyperactivated” NK cells with enhanced antitumoral activity and secreting factors that facilitated oncolytic virus spread throughout the tumor by disrupting the tumor architecture (105, 106). Survival benefit by this combination therapy was compromised when NK cells were depleted. Additionally, Cheema et al. could reduce regulatory T cell population in the tumor by arming an oHSV with the cytokine IL12 leading to increased survival in a murine glioblastome stem cell model. Survival benefit by additional expression of IL-12 was absent in athymic mouse indicating that antitumoral efficacy was T cell dependent (107). In contrast Treg depletion was demonstrated to have even a negative therapeutic effect on VSV therapy by relieving Treg-mediated suppression of antiviral immunity resulting in rapid clearance of the therapeutic vector (91).

However, the consequences of Treg depletion on long-term antitumoral T cell responses that can be induced by oncolytic virotherapy are not clear. Observations in classical infection models have shown that migratory activity of Tregs plays a central role in eliciting a protective immunity to viral infection (108). Consistent with a positive function of Tregs in shaping antigen-specific immune responses, we have observed that Treg depletion abrogated the effective antitumoral T cell induction by an oncolysis-assisted, antitumoral DC-vaccine (109). We could also show that immunosuppressive MDSC expand in Treg-depleted tumors, which may explain the failure of antitumoral T cell priming. Supporting an important role of Tregs in the priming of antigen-specific T cells, it has been described that Tregs can undergo a conversion under acute inflammatory conditions to adopt a T helper phenotype (110). Converted Tregs express proinflammatory cytokines and activate additional functions to provide effective help for triggering T cell responses against new antigens. These findings described above indicate that Tregs can essentially modulate the course of tumor therapy with oncolytic viruses. A supportive role of Treg depletion on virus spread and therapeutic efficacy of oncolysis is still unclear and possible consequences on induction of sustainable tumor-directed T cell responses require further investigations.

## Harnessing Oncolytic Virotherapy as Immunotherapy

Observations in immunocompromised xenografts have tempted to overestimate the cytolytic effects that are achievable in human patients. The situation in the immunocompetent host is completely different with positive and negative consequences for the therapeutic efficacy of virotherapy. Since it is known that T cell responses against cross-presented cellular antigens upon viral infections trigger innate immune receptor pathways such as TLRs and MyD88 (11, 12), investigations on corresponding antitumoral immunity have been intensively pursued in oncolytic virus applications in immunocompetent models. The use of oncolytic VSV in the B16-Ova model strikingly demonstrated that antitumoral effects completely depended on Type I IFN responses, which mediate both antiviral protection and antitumor therapy, whereas VSV-mediated therapy was abolished in MyD88^−/−^ mice (111). The relevance of both innate immune activation and subsequent triggering of adaptive responses was shown in experimental models with T cell depletion studies (10). Interesting observation have been reported using herpes simplex virus variants with different replicative properties. oHSV vectors that were more rapidly cleared from the tumor but induced higher levels of DAMPs resulted in best survival. This strongly indicates that replicative potency is not the dominating factor as believed before but emphasizes the impact of the initial immune induction (112), which needs to be considered in the rational designs of novel approaches aiming at increased antitumor immunity. DC are known to play a crucial role in the generation of tumor-directed T cell responses (113). First strategies on utilizing oncolytic virotherapy to engage intrinsic activity of DC were performed with an ICP34.5 deleted herpes simplex virus coding for GM-CSF (114). Tumor infection with this oncolytic virus led to regression and protected the mice against rechallenge with tumor cells. GM-CSF-expressing HSV then entered clinical development as OncoVexGM-CSF or T-Vec (14, 115). Furthermore, virus-encoded GM-CSF not only affected DCs, but also neutrophils which were shown to contribute to antitumor effects by a GM-CSF-expressing oncolytic measles virus in CD46 transgenic mice (70). The therapeutic benefit of engaging dendritic cell activity in virotherapeutic applications was confirmed using different cytokine setups. In a preclinical breast cancer model, systemic, and intratumoral delivery of a TRAIL-/E1A-expressing oncolytic adenovirus increased plasma levels of TNFα, IFNγ, and MCP-1, proinflammatory cytokines acting as maturation signals for DCs. Inclusion of FLT3L or GM-CSF-expressing adenovirus for expansion of DCs established systemic antitumor immunity and resulted in tumor elimination (116). We obtained consistent results in a mouse model of lung cancer using intratumoral delivery of an oncolytic Ad combined with vectors encoding FLT3L and MIP-1α. Tumor-directed T cells were significantly increased and improved tumor responses were obtained. However, adaptive immune responses against the viral vector were also strongly enhanced suggesting that the balance between tumor- and virus-directed immunity remains unaltered instead of generating a favorable tumor-directed response (117). Oncolytic viruses expressing cytokines for enhanced antigen cross presentation illustrate that virotherapy can be used as a tool for a generic *in situ* vaccination without the need for detailed information about specific tumor-specific antigens. However, the approach has limitations in shifting the predominant antiviral responses in favor of antitumoral responses.

## Oncolytic Virotherapy in DC-Vaccinations and Heterologous Prime-Boost Settings

For focusing the immune system during virotherapy on the tumor requires the incorporation of tumor-specific antigen targeting approaches into the therapeutic scheme. We have investigated this aspect by combining viral oncolysis and a tumor-directed DC-vaccine (117). In another study, it has been shown that a CCL5 (RANTES) expressing oncolytic vaccinia virus significantly improved the therapeutic efficacy of a tumor-directed DC-vaccine (118). In a further study, it was demonstrated that the application of a replicating adenovirus allowed for highly effective DC-vaccination, when the vaccine is administered exactly at the time of apparent virus-induced tumor inflammation (109). This approach induced potent cytotoxic T cell responses leading to significant tumor regression and complete eradication of lung colonies in an aggressive tumor model that was otherwise resistant to the DC-vaccine. A further promising direction is the development of oncolytic virus-based prime-boost strategies that express the tumor-antigen. In a heterologous treatment sequence with an adenoviral TAA-endoding vaccine and an oncolytic VSV tumor expressing the same antigen significantly enhanced tumor-directed CD8 T cell immune responses compared to single treatments. Heterologous priming worked in both directions (119, 120). This approach shifted the immune responses from viral antigens to tumor-antigens and reduced viral replication in healthy tissues thereby improving efficacy and safety. Interestingly, the magnitude of tumor-specific responses after combination therapy was even higher in tumor-bearing hosts compared to tumor-free mice indicating the need of infected tumor tissue for priming antitumoral T cell reponses (120). The same group could also demonstrate that heterologous boosting not only resulted in higher numbers but also in functionally superior T cells (121). A further interesting variation of prime-boost vaccinations comes from Brinkhoff and colleagues who elicited highest antitumoral responses when the boost step by an antigen-expressing infectious agent was preceded by a non-pathogenic prime using antigen-loaded PLGA-microspheres (122).

## Targeting the Tumor Antigenome and Mutanome by Oncolytic Virotherapy

The use of complete antigen libraries encoded by an oncolytic virus offers a promising approach to circumvent the limitations of antigen-specific vaccinations. In a preclinical study in prostate cancer, VSV-based cDNA libraries from xenogeneic healthy prostrate tissue were used for treatment of TC2 prostrate tumors. Application of VSVs coding for such a cDNA library [Altered Self-antigen and Epitope Library (ASEL)] cured established tumors after repetitive intravenous injections. The use of ASEL conferred significantly better protection against TC2 cells than a self-antigen library from normal mouse prostate tissue. Upon application of ASEL, a T_H_17 response was detectable and TC2 rejection was dependent on CD4 cells, but not on CD8 T cells or NK cells (123). A subsequent study from this group demonstrated that an approach of virus-encoded melanoma cDNA libraries can be used to identify tumor-associated antigens that have the ability to cure melanoma (124). Virus-expressed cDNA libraries were effective against melanoma thereby inducing only mild signs of autoimmunity. The xenogenic, altered self-source is a precondition for successful tumor treatment due to additional adjuvant effects compared to a library from an autologous self-source. Again, the antitumoral effect was correlated with a tumor-specific IL-17 response, which was in turn utilized to screen for cDNA-viruses that induced IL-17 memory for identification of tumor rejection antigens. After validation of IL-17 inducing clones, three VSV-encoded tumor-antigens were tested to treat established B16 tumors. Intriguingly, injection of a single VSV-clone or a pool of two VSV-clones did not show a therapeutic response, only the combination of all three VSV-clones cured melanoma tumors to a similar extent as the whole melanoma-library did. Although, it remains unclear why only all three different TAA-coding VSVs contribute to therapeutic effects, this finding suggest that applications targeting multiple antigens at the same time should be preferred in immunotherapeutic strategies. These studies establish a rational approach to identify novel tumor-targets for immunotherapy and establish an effective generic virus-based ASEL-vaccine for defined tumor entities.

To date, identification of novel tumor-antigens that can be addressed by targeted therapeutics appears to be a crucial step toward the establishment of clinically effective immunotherapies and toward induction of sustained adaptive T cell responses. In the past, antitumoral vaccine research has focused on finding non-mutated, tumor-associated antigens such as telomerase or MAGE, which can be found either in a relevant numbers of patients and/or across several entities to promise broad applicability. Disappointingly, corresponding vaccination approaches have so far delivered insufficient effects in the clinic (125). A limiting factor is that non-mutated tumor-antigens may not reflect essential molecular functions required for tumor cell survival promoting the generation of escape variants (126). Furthermore, T cell precursors against this type of antigens are subject to thymus selection and self-tolerance mechanisms thus limiting the number of required high-affinity T cell precursors that are essential for effective antitumoral T cell responses. In this regard, triggering T cells that recognize immunogenic neoepitopes reflecting tumor-associated mutated proteins could be a more promising alternative. Data from melanoma patients indicate that autologous T cell responses to tumors are predominantly directed to neoantigens (127). In murine models as well, tumor rejection responses were also primarily induced by altered-self antigens (128, 129). However, this would require individualized (personalized) molecular diagnosis and therapy. Individual (solid) human cancers usually harbor about 30 to more than hundred of protein-encoded mutations referred to as mutanome (129–131), which can be nowadays rapidly and cost-effectively analyzed by Next Generation Sequencing (NGS) technology. Using this method, non-synonymous single nucleotide variants (SNV) can be identified, representing promising candidates for immunotherapies, since single amino acid variations in corresponding epitopes can be processed and presented by MHC to T cells.

In a pioneering study targeting the mutanome by vaccinations, NGS was used for immunoepitope identification in B16F10 melanoma cells. Selected from 563 non-synonymous SNV candidates, the immunogenicity of 50 validated mutations was determined using corresponding peptide immunizations in mice. The authors showed that immune responses could be raised against 60% of these epitopes and the vaccinations against these predicted and validated epitopes successfully raised antitumoral adaptive immune responses and significantly slowed tumor-growth (132). This illustrates the great potential of this method in identification of neoepitopes. However, the observation is also astonishing since those epitopes should be per definition of low immunogenic nature. In clinically manifest tumors, the remaining epitope spectrum is the result of a dynamic process termed cancer immunoediting, which acts on nascent tumors via different immune cell types to protect against cancer development and shapes the tumor at the same time toward decreased immunogenicity (129). In the study by Castle and colleagues, the key for successful induction of immune responses to immunoedited tumor-epitopes by DC-vaccination is most likely attributable to the use of adjuvants, i.e., poly(I:C) in the B16F10 model. Oncolytic virotherapy is likewise a potent trigger of innate immune receptors and inflammation and could be an interesting tool that enables identification of inflammation induced neoepitope-directed T cell responses and to cooperate with tailored neoepitope-directed DC-vaccines. However, it will be a challenging task to identify neoepitope-specific T cell reactivities that are involved in tumor responses induced by oncolytic virotherapy.

## Oncolytic Virotherapy and Immune Checkpoint Blockade

The recent clinical success of immune checkpoint blockade (133) has confirmed the curative potential of tumor immunotherapies. Checkpoint blockade using ipilimumab, a CTLA-4-blocking monoclonal antibody, has shown promising results in a phase III study (134). Remarkably, responses seemed to include even complete cures, but only a small proportion of patients benefited from therapy. In a case study which described a patient with advanced melanoma experiencing tumor response under ipilimumab, neoepitope analysis by NGS and epitope prediction led to identification of a single ipilimumab-responsive neoepitope-specific CD8 T cell that increased fivefold under therapy and remained stable for a 10-month period (135). The fact, that only one epitope was triggered in a tumor displaying 448 potential T cell neoepitopes is remarkable but reflects that natural and thus immunoedited tumors are low immunogenic despite harboring a high number of mutations and may also explain why only small subgroups of patients respond to certain immunotherapies. Oncolytic viruses can serve as an ideal tool to augment tumor immunogenicity and could be ideally combined with immune checkpoint blockade. Gao and colleagues have investigated the application of a Her2/neu targeted oncolytic VSV in combination with a CTLA-4 antibody in mice bearing Her2/neu transgenic murine mammary tumors. This combination achieved cure in the majority of mice whereas the virotherapy alone only prolonged survival (136). Additionally, it has been tried to include an expression cassette for a CTLA-4-specific antibody into the backbone of an oncolytic Ad to enhance local concentrations and to avoid adverse events by systemic CTLA-4 inhibition (137). Recently, it has been reported that injection of oncolytic NDV in a preclinical model of B16 melanoma under CTLA-4 antibody treatment induces an inflammatory response in tumor tissue, leading to lymphocytic infiltration and antitumor effect in distant, non-virally injected tumors (138). Effective treatment induced activated CD4 and CD8 T cell infiltration in distant tumors and was dependent on CD8^+^ cells, natural killer cells, and type I IFNs. Overcoming systemic resistance to immune checkpoint blockade by oncolytic virotherapy moreover led to protection from tumor rechallenge in poorly immunogenic tumors, even in a cell line refractory to NDV-mediated lysis. An alternative to checkpoint blockade is the direct activation of costimulation using oncolytic viruses expressing the costimulatory CD40L (53). Further approaches used oncolytic vaccinia viruses expressing the ligand for the costimulatory receptor 4-1BB (CD137) that achieved maximum antitumoral efficacy in lymphodepleted hosts (139). Strong antitumoral immune responses were also elicited by combining oncolytic vaccinia virus with systemic application of a 4-1BB agonistic antibody (140). An interesting immune checkpoint that has not yet been investigated with virotherapy is PD-1/PD-L1. PD-1/PD-L1-blocking antibodies are in a very promising clinical development (141). PD-1/PD-L1 inhibition primarily activates antigen-experienced T cell responses in the periphery, thus providing a mechanism that could be promising to combine with virotherapeutic treatments.

## Perspective: Oncolytic Virotherapy in Multimodale Therapies

**Figure 2:**
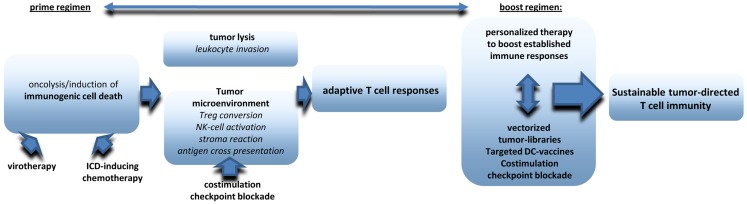
**The figure provides an overview on critical components to be included in multimodale virotherapy-based therapies that work like prime-boost strategies**.

There is increasing evidence that oncolytic virotherapy shows antitumoral efficacy in clinical application even as monotherapy. However, most preclinical data suggest that virotherapy can be ideally combined with other treatment options to raise significant therapeutic synergies on several levels (see also an overview in Figure 2). First of all, oncolytic virus treatment needs to be integrated in combined tumor-treatments leading to optimized induction of ICD. Excellent reviews already exist on this aspect (16, 142, 143). Next step should be additional measures that amplify, and prolong antitumoral immune responses. First data obtained in humans and in murine melanoma models suggest significant synergies when systemic immunotherapies, such as ipilimumab and virotherapy are combined in a well-coordinated manner (138, 144). A very promising but clinically challenging point will be the combination of viral oncolysis with surgical removal of the tumor. Finally, it still needs further investigations to establish follow-up therapies that work like classical boost strategies and may also pick up personalized approaches such as NGS of tumors, epitope prediction and and immunoanalysis in treated patients. Multimodal therapy schemes will be a clue to establish virotherapy in the clinic.

## Conflict of Interest Statement

The authors declare that the research was conducted in the absence of any commercial or financial relationships that could be construed as a potential conflict of interest.

## References

[1] Russell SJ Peng KW Bell JC Oncolytic virotherapy. Nat Biotechnol (2012) 30:658–70 10.1038/nbt.2287 22781695 PMC3888062

[2] Aghi M Martuza RL Oncolytic viral therapies – the clinical experience. Oncogene (2005) 24:7802–16 10.1038/sj.onc.1209037 16299539

[3] Heise C Sampson-Johannes A Williams A McCormick F Von Hoff DD Kirn DH ONYX-015, an E1B gene-attenuated adenovirus, causes tumor-specific cytolysis and antitumoral efficacy that can be augmented by standard chemotherapeutic agents. Nat Med (1997) 3:639–45 10.1038/nm0697-639 9176490

[4] Garber K China approves world’s first oncolytic virus therapy for cancer treatment. J Natl Cancer Inst (2006) 98:298–300 10.1093/jnci/djj111 16507823

[5] Smith E Breznik J Lichty BD Strategies to enhance viral penetration of solid tumors. Hum Gene Ther (2011) 22:1053–60 10.1089/hum.2010.227 21443415

[6] Prestwich RJ Errington F Diaz RM Pandha HS Harrington KJ Melcher AA The case of oncolytic viruses versus the immune system: waiting on the judgment of Solomon. Hum Gene Ther (2009) 20:1119–32 10.1089/hum.2009.135 19630549 PMC2829276

[7] Wongthida P Diaz RM Galivo F Kottke T Thompson J Pulido J Type III IFN interleukin-28 mediates the antitumor efficacy of oncolytic virus VSV in immune-competent mouse models of cancer. Cancer Res (2010) 70:4539–49 10.1158/0008-5472.CAN-09-4658 20484025 PMC3896099

[8] Breitbach CJ Arulanandam R De SN Thorne SH Patt R Daneshmand M Oncolytic vaccinia virus disrupts tumor-associated vasculature in humans. Cancer Res (2013) 73:1265–75 10.1158/0008-5472.CAN-12-2687 23393196

[9] Breitbach CJ Paterson JM Lemay CG Falls TJ McGuire A Parato KA Targeted inflammation during oncolytic virus therapy severely compromises tumor blood flow. Mol Ther (2007) 15:1686–93 10.1038/sj.mt.6300215 17579581

[10] Sobol PT Boudreau JE Stephenson K Wan Y Lichty BD Mossman KL Adaptive antiviral immunity is a determinant of the therapeutic success of oncolytic virotherapy. Mol Ther (2011) 19:335–44 10.1038/mt.2010.264 21119618 PMC3034857

[11] Palliser D Ploegh H Boes M Myeloid differentiation factor 88 is required for cross-priming in vivo. J Immunol (2004) 172:3415–21 10.4049/jimmunol.172.6.3415 15004140

[12] Schulz O Diebold SS Chen M Naslund TI Nolte MA Alexopoulou L Toll-like receptor 3 promotes cross-priming to virus-infected cells. Nature (2005) 433:887–92 10.1038/nature03326 15711573

[13] Park BH Hwang T Liu TC Sze DY Kim JS Kwon HC Use of a targeted oncolytic poxvirus, JX-594, in patients with refractory primary or metastatic liver cancer: a phase I trial. Lancet Oncol (2008) 9:533–42 10.1016/S1470-2045(08)70107-4 18495536

[14] Kaufman HL Bines SD OPTIM trial: a Phase III trial of an oncolytic herpes virus encoding GM-CSF for unresectable stage III or IV melanoma. Future Oncol (2010) 6:941–9 10.2217/fon.10.66 20528232

[15] Thomas MA Spencer JF Toth K Sagartz JE Phillips NJ Wold WS Immunosuppression enhances oncolytic adenovirus replication and antitumor efficacy in the Syrian hamster model. Mol Ther (2008) 16:1665–73 10.1038/mt.2008.162 18665155 PMC3437752

[16] Bartlett DL Liu Z Sathaiah M Ravindranathan R Guo Z He Y Oncolytic viruses as therapeutic cancer vaccines. Mol Cancer (2013) 12:103 10.1186/1476-4598-12-103 24020520 PMC3847443

[17] Kepp O Senovilla L Galluzzi L Panaretakis T Tesniere A Schlemmer F Viral subversion of immunogenic cell death. Cell Cycle (2009) 8:860–9 10.4161/cc.8.6.7939 19221507

[18] Lousberg EL Diener KR Brown MP Hayball JD Innate immune recognition of poxviral vaccine vectors. Expert Rev Vaccines (2011) 10:1435–49 10.1586/erv.11.121 21988308

[19] Kroemer G Galluzzi L Vandenabeele P Abrams J Alnemri ES Baehrecke EH Classification of cell death: recommendations of the Nomenclature Committee on Cell Death 2009. Cell Death Differ (2009) 16:3–11 10.1038/cdd.2008.150 18846107 PMC2744427

[20] Fadok VA Bratton DL Konowal A Freed PW Westcott JY Henson PM Macrophages that have ingested apoptotic cells in vitro inhibit proinflammatory cytokine production through autocrine/paracrine mechanisms involving TGF-beta, PGE2, and PAF. J Clin Invest (1998) 101:890–8 10.1172/JCI1112 9466984 PMC508637

[21] Fadok VA Bratton DL Guthrie L Henson PM Differential effects of apoptotic versus lysed cells on macrophage production of cytokines: role of proteases. J Immunol (2001) 166:6847–54 10.4049/jimmunol.166.11.6847 11359844

[22] Tesniere A Schlemmer F Boige V Kepp O Martins I Ghiringhelli F Immunogenic death of colon cancer cells treated with oxaliplatin. Oncogene (2010) 29:482–91 10.1038/onc.2009.356 19881547

[23] Casares N Pequignot MO Tesniere A Ghiringhelli F Roux S Chaput N Caspase-dependent immunogenicity of doxorubicin-induced tumor cell death. J Exp Med (2005) 202:1691–701 10.1084/jem.20050915 16365148 PMC2212968

[24] Kroemer G Galluzzi L Kepp O Zitvogel L Immunogenic cell death in cancer therapy. Annu Rev Immunol (2013) 31:51–72 10.1146/annurev-immunol-032712-100008 23157435

[25] Abou E Hassan MA van Meulen-Muileman I Abbas S Kruyt FA Conditionally replicating adenoviruses kill tumor cells via a basic apoptotic machinery-independent mechanism that resembles necrosis-like programmed cell death. J Virol (2004) 78:12243–51 10.1128/JVI.78.22.12243-12251.2004 15507611 PMC525077

[26] Whilding LM Archibald KM Kulbe H Balkwill FR Oberg D McNeish IA Vaccinia virus induces programmed necrosis in ovarian cancer cells. Mol Ther (2013) 21:2074–86 10.1038/mt.2013.195 23985697 PMC3831043

[27] Vandenabeele P Galluzzi L Vanden Berghe T Kroemer G Molecular mechanisms of necroptosis: an ordered cellular explosion. Nat Rev Mol Cell Biol (2010) 11:700–14 10.1038/nrm2970 20823910

[28] Oberst A Dillon CP Weinlich R McCormick LL Fitzgerald P Pop C Catalytic activity of the caspase-8-FLIP(L) complex inhibits RIPK3-dependent necrosis. Nature (2011) 471:363–7 10.1038/nature09852 21368763 PMC3077893

[29] Cai Z Jitkaew S Zhao J Chiang HC Choksi S Liu J Plasma membrane translocation of trimerized MLKL protein is required for TNF-induced necroptosis. Nat Cell Biol (2014) 16:55–65 10.1038/ncb2883 24316671 PMC8369836

[30] Mocarski ES Kaiser WJ Livingston-Rosanoff D Upton JW Daley-Bauer LP True grit: programmed necrosis in antiviral host defense, inflammation, and immunogenicity. J Immunol (2014) 192:2019–26 10.4049/jimmunol.1302426 24563506 PMC3934821

[31] Kaiser WJ Upton JW Long AB Livingston-Rosanoff D Daley-Bauer LP Hakem R RIP3 mediates the embryonic lethality of caspase-8-deficient mice. Nature (2011) 471:368–72 10.1038/nature09857 21368762 PMC3060292

[32] Cho YS Challa S Moquin D Genga R Ray TD Guildford M Phosphorylation-driven assembly of the RIP1-RIP3 complex regulates programmed necrosis and virus-induced inflammation. Cell (2009) 137:1112–23 10.1016/j.cell.2009.05.037 19524513 PMC2727676

[33] Upton JW Kaiser WJ Mocarski ES Virus inhibition of RIP3-dependent necrosis. Cell Host Microbe (2010) 7:302–13 10.1016/j.chom.2010.03.006 20413098 PMC4279434

[34] McNamara CR Ahuja R Osafo-Addo AD Barrows D Kettenbach A Skidan I Akt Regulates TNFalpha synthesis downstream of RIP1 kinase activation during necroptosis. PLoS One (2013) 8:e56576 10.1371/journal.pone.0056576 23469174 PMC3585731

[35] Boozari B Mundt B Woller N Struver N Gurlevik E Schache P Antitumoural immunity by virus-mediated immunogenic apoptosis inhibits metastatic growth of hepatocellular carcinoma. Gut (2010) 59:1416–26 10.1136/gut.2009.196519 20675696

[36] Weiss R Sachet M Zinngrebe J Aschacher T Krainer M Hegedus B IL-24 sensitizes tumor cells to TLR3-mediated apoptosis. Cell Death Differ (2013) 20:823–33 10.1038/cdd.2013.15 23449395 PMC3647237

[37] English L Chemali M Duron J Rondeau C Laplante A Gingras D Autophagy enhances the presentation of endogenous viral antigens on MHC class I molecules during HSV-1 infection. Nat Immunol (2009) 10:480–7 10.1038/ni.1720 19305394 PMC3885169

[38] Ito H Aoki H Kuhnel F Kondo Y Kubicka S Wirth T Autophagic cell death of malignant glioma cells induced by a conditionally replicating adenovirus. J Natl Cancer Inst (2006) 98:625–36 10.1093/jnci/djj161 16670388

[39] Jiang H Gomez-Manzano C Aoki H Alonso MM Kondo S McCormick F Examination of the therapeutic potential of Delta-24-RGD in brain tumor stem cells: role of autophagic cell death. J Natl Cancer Inst (2007) 99:1410–4 10.1093/jnci/djm102 17848677

[40] Meng C Zhou Z Jiang K Yu S Jia L Wu Y Newcastle disease virus triggers autophagy in U251 glioma cells to enhance virus replication. Arch Virol (2012) 157:1011–8 10.1007/s00705-012-1270-6 22398914 PMC7087167

[41] Sun Y Yu S Ding N Meng C Meng S Zhang S Autophagy benefits the replication of Newcastle disease virus in chicken cells and tissues. J Virol (2014) 88:525–37 10.1128/JVI.01849-13 24173218 PMC3911706

[42] Yokoyama T Iwado E Kondo Y Aoki H Hayashi Y Georgescu MM Autophagy-inducing agents augment the antitumor effect of telerase-selve oncolytic adenovirus OBP-405 on glioblastoma cells. Gene Ther (2008) 15:1233–9 10.1038/gt.2008.98 18580968

[43] Xia M Gonzalez P Li C Meng G Jiang A Wang H Mitophagy enhances oncolytic measles virus replication by mitigating DDX58/RIG-I-like receptor signaling. J Virol (2014) 88:5152–64 10.1128/JVI.03851-13 24574393 PMC3993837

[44] Tang D Kang R Coyne CB Zeh HJ Lotze MT PAMPs and DAMPs: signal 0s that spur autophagy and immunity. Immunol Rev (2012) 249:158–75 10.1111/j.1600-065X.2012.01146.x 22889221 PMC3662247

[45] Matzinger P Tolerance, danger, and the extended family. Annu Rev Immunol (1994) 12:991–1045 10.1146/annurev.immunol.12.1.991 8011301

[46] Elliott MR Chekeni FB Trampont PC Lazarowski ER Kadl A Walk SF Nucleotides released by apoptotic cells act as a find-me signal to promote phagocytic clearance. Nature (2009) 461:282–6 10.1038/nature08296 19741708 PMC2851546

[47] Chekeni FB Elliott MR Sandilos JK Walk SF Kinchen JM Lazarowski ER Pannexin 1 channels mediate ‘find-me’ signal release and membrane permeability during apoptosis. Nature (2010) 467:863–7 10.1038/nature09413 20944749 PMC3006164

[48] Ghiringhelli F Apetoh L Tesniere A Aymeric L Ma Y Ortiz C Activation of the NLRP3 inflammasome in dendritic cells induces IL-1beta-dependent adaptive immunity against tumors. Nat Med (2009) 15:1170–8 10.1038/nm.2028 19767732

[49] Liikanen I Ahtiainen L Hirvinen ML Bramante S Cerullo V Nokisalmi P Oncolytic adenovirus with temozolomide induces autophagy and antitumor immune responses in cancer patients. Mol Ther (2013) 21:1212–23 10.1038/mt.2013.51 23546299 PMC3681222

[50] Miyamoto S Inoue H Nakamura T Yamada M Sakamoto C Urata Y Coxsackievirus B3 is an oncolytic virus with immunostimulatory properties that is active against lung adenocarcinoma. Cancer Res (2012) 72:2609–21 10.1158/0008-5472.CAN-11-3185 22461509

[51] Garg AD Krysko DV Verfaillie T Kaczmarek A Ferreira GB Marysael T A novel pathway combining calreticulin exposure and ATP secretion in immunogenic cancer cell death. EMBO J (2012) 31:1062–79 10.1038/emboj.2011.497 22252128 PMC3298003

[52] Obeid M Tesniere A Panaretakis T Tufi R Joza N van Endert P Ecto-calreticulin in immunogenic chemotherapy. Immunol Rev (2007) 220:22–34 10.1111/j.1600-065X.2007.00567.x 17979837

[53] Diaconu I Cerullo V Hirvinen ML Escutenaire S Ugolini M Pesonen SK Immune response is an important aspect of the antitumor effect produced by a CD40L-encoding oncolytic adenovirus. Cancer Res (2012) 72:2327–38 10.1158/0008-5472.CAN-11-2975 22396493

[54] Scaffidi P Misteli T Bianchi ME Release of chromatin protein HMGB1 by necrotic cells triggers inflammation. Nature (2002) 418:191–5 10.1038/nature00858 12110890

[55] Kepp O Menger L Vacchelli E Locher C Adjemian S Yamazaki T Crosstalk between ER stress and immunogenic cell death. Cytokine Growth Factor Rev (2013) 24:311–8 10.1016/j.cytogfr.2013.05.001 23787159

[56] Donnelly OG Errington-Mais F Steele L Hadac E Jennings V Scott K Measles virus causes immunogenic cell death in human melanoma. Gene Ther (2013) 20:7–15 10.1038/gt.2011.205 22170342 PMC3378495

[57] Guo ZS Naik A O’Malley ME Popovic P Demarco R Hu Y The enhanced tumor selectivity of an oncolytic vaccinia lacking the host range and antiapoptosis genes SPI-1 and SPI-2. Cancer Res (2005) 65:9991–8 10.1158/0008-5472.CAN-05-1630 16267024

[58] Moehler M Zeidler M Schede J Rommelaere J Galle PR Cornelis JJ Oncolytic parvovirus H1 induces release of heat-shock protein HSP72 in susceptible human tumor cells but may not affect primary immune cells. Cancer Gene Ther (2003) 10:477–80 10.1038/sj.cgt.7700591 12768193

[59] Kono H Chen CJ Ontiveros F Rock KL Uric acid promotes an acute inflammatory response to sterile cell death in mice. J Clin Invest (2010) 120:1939–49 10.1172/JCI40124 20501947 PMC2877935

[60] Endo Y Sakai R Ouchi M Onimatsu H Hioki M Kagawa S Virus-mediated oncolysis induces danger signal and stimulates cytotoxic T-lymphocyte activity via proteasome activator upregulation. Oncogene (2008) 27:2375–81 10.1038/sj.onc.1210884 17982491

[61] Gamrekelashvili J Kapanadze T Han M Wissing J Ma C Jaensch L Peptidases released by necrotic cells control CD8+ T cell cross-priming. J Clin Invest (2013) 123:4755–68 10.1172/JCI65698 24216478 PMC3809774

[62] Mahoney DJ Lefebvre C Allan K Brun J Sanaei CA Baird S Virus-tumor interactome screen reveals ER stress response can reprogram resistant cancers for oncolytic virus-triggered caspase-2 cell death. Cancer Cell (2011) 20:443–56 10.1016/j.ccr.2011.09.005 22014571

[63] Kelly KR Espitia CM Mahalingam D Oyajobi BO Coffey M Giles FJ Reovirus therapy stimulates endoplasmic reticular stress, NOXA induction, and augments bortezomib-mediated apoptosis in multiple myeloma. Oncogene (2012) 31:3023–38 10.1038/onc.2011.478 22002308

[64] Yoo JY Hurwitz BS Bolyard C Yu JG Zhang J Selvendiran K Bortezomib-induced unfolded protein response increases oncolytic HSV-1 replication resulting in synergistic, anti-tumor effects. Clin Cancer Res (2014). 10.1158/1078-0432.CCR-14-0553 24815720 PMC4132885

[65] Prestwich RJ Errington F Ilett EJ Morgan RS Scott KJ Kottke T Tumor infection by oncolytic reovirus primes adaptive antitumor immunity. Clin Cancer Res (2008) 14:7358–66 10.1158/1078-0432.CCR-08-0831 19010851 PMC2701231

[66] McKee TD Grandi P Mok W Alexandrakis G Insin N Zimmer JP Degradation of fibrillar collagen in a human melanoma xenograft improves the efficacy of an oncolytic herpes simplex virus vector. Cancer Res (2006) 66:2509–13 10.1158/0008-5472.CAN-05-2242 16510565

[67] Mok W Boucher Y Jain RK Matrix metalloproteinases-1 and -8 improve the distribution and efficacy of an oncolytic virus. Cancer Res (2007) 67:10664–8 10.1158/0008-5472.CAN-07-3107 18006807

[68] Di Paolo NC Miao EA Iwakura Y Murali-Krishna K Aderem A Flavell RA Virus binding to a plasma membrane receptor triggers interleukin-1 alpha-mediated proinflammatory macrophage response in vivo. Immunity (2009) 31:110–21 10.1016/j.immuni.2009.04.015 19576795 PMC2759279

[69] Di Paolo NC Doronin K Baldwin LK Papayannopoulou T Shayakhmetov DM The transcription factor IRF3 triggers "defensive suicide" necrosis in response to viral and bacterial pathogens. Cell Rep (2013) 3:1840–6 10.1016/j.celrep.2013.05.025 23770239 PMC3718285

[70] Grote D Cattaneo R Fielding AK Neutrophils contribute to the measles virus-induced antitumor effect: enhancement by granulocyte macrophage colony-stimulating factor expression. Cancer Res (2003) 63:6463–8 14559838

[71] Iankov ID Allen C Federspiel MJ Myers RM Peng KW Ingle JN Expression of immunomodulatory neutrophil-activating protein of Helicobacter pylori enhances the antitumor activity of oncolytic measles virus. Mol Ther (2012) 20:1139–47 10.1038/mt.2012.4 22334023 PMC3369290

[72] Zhang Y Patel B Dey A Ghorani E Rai L Elham M Attenuated, oncolytic, but not wild-type measles virus infection has pleiotropic effects on human neutrophil function. J Immunol (2012) 188:1002–10 10.4049/jimmunol.1102262 22180616

[73] Ikeda K Ichikawa T Wakimoto H Silver JS Deisboeck TS Finkelstein D Oncolytic virus therapy of multiple tumors in the brain requires suppression of innate and elicited antiviral responses. Nat Med (1999) 5:881–7 10.1038/11320 10426310

[74] Altomonte J Wu L Chen L Meseck M Ebert O Garcia-Sastre A Exponential enhancement of oncolytic vesicular stomatitis virus potency by vector-mediated suppression of inflammatory responses in vivo. Mol Ther (2008) 16:146–53 10.1038/sj.mt.6300343 18071337 PMC2930752

[75] Alvarez-Breckenridge CA Yu J Price R Wojton J Pradarelli J Mao H NK cells impede glioblastoma virotherapy through NKp30 and NKp46 natural cytotoxicity receptors. Nat Med (2012) 18:1827–34 10.1038/nm.3013 23178246 PMC3668784

[76] Fulci G Breymann L Gianni D Kurozomi K Rhee SS Yu J Cyclophosphamide enhances glioma virotherapy by inhibiting innate immune responses. Proc Natl Acad Sci U S A (2006) 103:12873–8 10.1073/pnas.0605496103 16908838 PMC1568940

[77] Berchtold S Lampe J Weiland T Smirnow I Schleicher S Handgretinger R Innate immune defense defines susceptibility of sarcoma cells to measles vaccine virus-based oncolysis. J Virol (2013) 87:3484–501 10.1128/JVI.02106-12 23302892 PMC3592150

[78] Motz GT Coukos G Deciphering and reversing tumor immune suppression. Immunity (2013) 39:61–73 10.1016/j.immuni.2013.07.005 23890064 PMC3782392

[79] Biswas SK Mantovani A Macrophage plasticity and interaction with lymphocyte subsets: cancer as a paradigm. Nat Immunol (2010) 11:889–96 10.1038/ni.1937 20856220

[80] Sica A Mantovani A Macrophage plasticity and polarization: in vivo veritas. J Clin Invest (2012) 122:787–95 10.1172/JCI59643 22378047 PMC3287223

[81] Fulci G Dmitrieva N Gianni D Fontana EJ Pan X Lu Y Depletion of peripheral macrophages and brain microglia increases brain tumor titers of oncolytic viruses. Cancer Res (2007) 67:9398–406 10.1158/0008-5472.CAN-07-1063 17909049 PMC2850558

[82] Fridlender ZG Sun J Kim S Kapoor V Cheng G Ling L Polarization of tumor-associated neutrophil phenotype by TGF-beta: "N1" versus "N2" TAN. Cancer Cell (2009) 16:183–94 10.1016/j.ccr.2009.06.017 19732719 PMC2754404

[83] Diaz-Montero CM Salem ML Nishimura MI Garrett-Mayer E Cole DJ Montero AJ Increased circulating myeloid-derived suppressor cells correlate with clinical cancer stage, metastatic tumor burden, and doxorubicin-cyclophosphamide chemotherapy. Cancer Immunol Immunother (2009) 58:49–59 10.1007/s00262-008-0523-4 18446337 PMC3401888

[84] Pedroza-Gonzalez A Verhoef C Ijzermans JN Peppelenbosch MP Kwekkeboom J Verheij J Activated tumor-infiltrating CD4+ regulatory T cells restrain antitumor immunity in patients with primary or metastatic liver cancer. Hepatology (2013) 57:183–94 10.1002/hep.26013 22911397

[85] Zou W Immunosuppressive networks in the tumour environment and their therapeutic relevance. Nat Rev Cancer (2005) 5:263–74 10.1038/nrc1586 15776005

[86] Suzuki E Kapoor V Jassar AS Kaiser LR Albelda SM Gemcitabine selectively eliminates splenic Gr-1+/CD11b+ myeloid suppressor cells in tumor-bearing animals and enhances antitumor immune activity. Clin Cancer Res (2005) 11:6713–21 10.1158/1078-0432.CCR-05-0883 16166452

[87] Esaki S Goshima F Kimura H Murakami S Nishiyama Y Enhanced antitumoral activity of oncolytic herpes simplex virus with gemcitabine using colorectal tumor models. Int J Cancer (2013) 132:1592–601 10.1002/ijc.27823 22949155

[88] Coca S Perez-Piqueras J Martinez D Colmenarejo A Saez MA Vallejo C The prognostic significance of intratumoral natural killer cells in patients with colorectal carcinoma. Cancer (1997) 79:2320–8 10.1002/(SICI)1097-0142(19970615)79:12<3C2320::AID-CNCR5>3E3.0.CO;2-P 9191519

[89] Biron CA Nguyen KB Pien GC Cousens LP Salazar-Mather TP Natural killer cells in antiviral defense: function and regulation by innate cytokines. Annu Rev Immunol (1999) 17:189–220 10.1146/annurev.immunol.17.1.189 10358757

[90] Altomonte J Wu L Meseck M Chen L Ebert O Garcia-Sastre A Enhanced oncolytic potency of vesicular stomatitis virus through vector-mediated inhibition of NK and NKT cells. Cancer Gene Ther (2009) 16:266–78 10.1038/cgt.2008.74 18846115 PMC2924743

[91] Diaz RM Galivo F Kottke T Wongthida P Qiao J Thompson J Oncolytic immunovirotherapy for melanoma using vesicular stomatitis virus. Cancer Res (2007) 67:2840–8 10.1158/0008-5472.CAN-06-3974 17363607

[92] Gujar SA Pan DA Marcato P Garant KA Lee PW Oncolytic virus-initiated protective immunity against prostate cancer. Mol Ther (2011) 19:797–804 10.1038/mt.2010.297 21245852 PMC3070098

[93] Rintoul JL Lemay CG Tai LH Stanford MM Falls TJ de Souza CT ORFV: a novel oncolytic and immune stimulating parapoxvirus therapeutic. Mol Ther (2012) 20:1148–57 10.1038/mt.2011.301 22273579 PMC3369287

[94] Tai LH de Souza CT Belanger S Ly L Alkayyal AA Zhang J Preventing postoperative metastatic disease by inhibiting surgery-induced dysfunction in natural killer cells. Cancer Res (2013) 73:97–107 10.1158/0008-5472.CAN-12-1993 23090117

[95] Tai LH Zhang J Scott KJ de Souza CT Alkayyal AA Ananth AA Perioperative influenza vaccination reduces postoperative metastatic disease by reversing surgery-induced dysfunction in natural killer cells. Clin Cancer Res (2013) 19:5104–15 10.1158/1078-0432.CCR-13-0246 23881927

[96] Li H Zeng Z Fu X Zhang X Coadministration of a herpes simplex virus-2 based oncolytic virus and cyclophosphamide produces a synergistic antitumor effect and enhances tumor-specific immune responses. Cancer Res (2007) 67:7850–5 10.1158/0008-5472.CAN-07-1087 17699791

[97] Myers RM Greiner SM Harvey ME Griesmann G Kuffel MJ Buhrow SA Preclinical pharmacology and toxicology of intravenous MV-NIS, an oncolytic measles virus administered with or without cyclophosphamide. Clin Pharmacol Ther (2007) 82:700–10 10.1038/sj.clpt.6100409 17971816 PMC2769566

[98] Zhang J Tai LH Ilkow CS Alkayyal AA Ananth AA de Souza CT Maraba MG1 virus enhances natural killer cell function via conventional dendritic cells to reduce postoperative metastatic disease. Mol Ther (2014) 22:1320–32 10.1038/mt.2014.60 24695102 PMC4088996

[99] Srivastava RM Lee SC Andrade Filho PA Lord CA Jie HB Davidson HC Cetuximab-activated natural killer and dendritic cells collaborate to trigger tumor antigen-specific T-cell immunity in head and neck cancer patients. Clin Cancer Res (2013) 19:1858–72 10.1158/1078-0432.CCR-12-2426 23444227 PMC3640274

[100] Kim MK Breitbach CJ Moon A Heo J Lee YK Cho M Oncolytic and immunotherapeutic vaccinia induces antibody-mediated complement-dependent cancer cell lysis in humans. Sci Transl Med (2013) 5:185ra63 10.1126/scitranslmed.3005361 23677592

[101] Gao Q Qiu SJ Fan J Zhou J Wang XY Xiao YS Intratumoral balance of regulatory and cytotoxic T cells is associated with prognosis of hepatocellular carcinoma after resection. J Clin Oncol (2007) 25:2586–93 10.1200/JCO.2006.09.4565 17577038

[102] Walker LS Treg and CTLA-4: two intertwining pathways to immune tolerance. J Autoimmun (2013) 45:49–57 10.1016/j.jaut.2013.06.006 23849743 PMC3989116

[103] Lanteri MC O’Brien KM Purtha WE Cameron MJ Lund JM Owen RE Tregs control the development of symptomatic West Nile virus infection in humans and mice. J Clin Invest (2009) 119:3266–77 10.1172/JCI39387 19855131 PMC2769173

[104] Oldenhove G Bouladoux N Wohlfert EA Hall JA Chou D Dos SL Decrease of Foxp3+ Treg cell number and acquisition of effector cell phenotype during lethal infection. Immunity (2009) 31:772–86 10.1016/j.immuni.2009.10.001 19896394 PMC2814877

[105] Kottke T Galivo F Wongthida P Diaz RM Thompson J Jevremovic D Treg depletion-enhanced IL-2 treatment facilitates therapy of established tumors using systemically delivered oncolytic virus. Mol Ther (2008) 16:1217–26 10.1038/mt.2008.83 18431359 PMC2729455

[106] Kottke T Thompson J Diaz RM Pulido J Willmon C Coffey M Improved systemic delivery of oncolytic reovirus to established tumors using preconditioning with cyclophosphamide-mediated Treg modulation and interleukin-2. Clin Cancer Res (2009) 15:561–9 10.1158/1078-0432.CCR-08-1688 19147761 PMC3046733

[107] Cheema TA Wakimoto H Fecci PE Ning J Kuroda T Jeyaretna DS Multifaceted oncolytic virus therapy for glioblastoma in an immunocompetent cancer stem cell model. Proc Natl Acad Sci U S A (2013) 110:12006–11 10.1073/pnas.1307935110 23754388 PMC3718117

[108] Lund JM Hsing L Pham TT Rudensky AY Coordination of early protective immunity to viral infection by regulatory T cells. Science (2008) 320:1220–4 10.1126/science.1155209 18436744 PMC2519146

[109] Woller N Knocke S Mundt B Gurlevik E Struver N Kloos A Virus-induced tumor inflammation facilitates effective DC cancer immunotherapy in a Treg-dependent manner in mice. J Clin Invest (2011) 121:2570–82 10.1172/JCI45585 21646722 PMC3223834

[110] Sharma MD Hou DY Baban B Koni PA He Y Chandler PR Reprogrammed foxp3(+) regulatory T cells provide essential help to support cross-presentation and CD8(+) T cell priming in naive mice. Immunity (2010) 33:942–54 10.1016/j.immuni.2010.11.022 21145762 PMC3032429

[111] Wongthida P Diaz RM Galivo F Kottke T Thompson J Melcher A VSV oncolytic virotherapy in the B16 model depends upon intact MyD88 signaling. Mol Ther (2011) 19:150–8 10.1038/mt.2010.225 20959810 PMC3017452

[112] Workenhe ST Simmons G Pol JG Lichty BD Halford WP Mossman KL Immunogenic HSV-mediated oncolysis shapes the antitumor immune response and contributes to therapeutic efficacy. Mol Ther (2014) 22:123–31 10.1038/mt.2013.238 24343053 PMC3978812

[113] Melief CJ Cancer immunotherapy by dendritic cells. Immunity (2008) 29:372–83 10.1016/j.immuni.2008.08.004 18799145

[114] Liu BL Robinson M Han ZQ Branston RH English C Reay P ICP34.5 deleted herpes simplex virus with enhanced oncolytic, immune stimulating, and anti-tumour properties. Gene Ther (2003) 10:292–303 10.1038/sj.gt.3301885 12595888

[115] Hu JC Coffin RS Davis CJ Graham NJ Groves N Guest PJ A phase I study of OncoVEXGM-CSF, a second-generation oncolytic herpes simplex virus expressing granulocyte macrophage colony-stimulating factor. Clin Cancer Res (2006) 12:6737–47 10.1158/1078-0432.CCR-06-0759 17121894

[116] Bernt KM Ni S Tieu AT Lieber A Assessment of a combined, adenovirus-mediated oncolytic and immunostimulatory tumor therapy. Cancer Res (2005) 65:4343–52 10.1158/0008-5472.CAN-04-3527 15899826

[117] Edukulla R Woller N Mundt B Knocke S Gurlevik E Saborowski M Antitumoral immune response by recruitment and expansion of dendritic cells in tumors infected with telomerase-dependent oncolytic viruses. Cancer Res (2009) 69:1448–58 10.1158/0008-5472.CAN-08-1160 19190348

[118] Li J O’Malley M Urban J Sampath P Guo ZS Kalinski P Chemokine expression from oncolytic vaccinia virus enhances vaccine therapies of cancer. Mol Ther (2011) 19:650–7 10.1038/mt.2010.312 21266959 PMC3070102

[119] Bridle BW Boudreau JE Lichty BD Brunelliere J Stephenson K Koshy S Vesicular stomatitis virus as a novel cancer vaccine vector to prime antitumor immunity amenable to rapid boosting with adenovirus. Mol Ther (2009) 17:1814–21 10.1038/mt.2009.154 19603003 PMC2835010

[120] Bridle BW Stephenson KB Boudreau JE Koshy S Kazdhan N Pullenayegum E Potentiating cancer immunotherapy using an oncolytic virus. Mol Ther (2010) 18:1430–9 10.1038/mt.2010.98 20551919 PMC2927075

[121] Bridle BW Clouthier D Zhang L Pol J Chen L Lichty BD Oncolytic vesicular stomatitis virus quantitatively and qualitatively improves primary CD8 T-cell responses to anticancer vaccines. Oncoimmunology (2013) 2:e26013 10.4161/onci.26013 24083086 PMC3782525

[122] Brinkhoff B Ostroumov D Heemcke J Woller N Gurlevik E Manns MP Microsphere priming facilitates induction of potent therapeutic T-cell immune responses against autochthonous liver cancers. Eur J Immunol (2014) 44:1213–24 10.1002/eji.201343794 24338782

[123] Kottke T Errington F Pulido J Galivo F Thompson J Wongthida P Broad antigenic coverage induced by vaccination with virus-based cDNA libraries cures established tumors. Nat Med (2011) 17:854–9 10.1038/nm.2390 21685898 PMC3918897

[124] Pulido J Kottke T Thompson J Galivo F Wongthida P Diaz RM Using virally expressed melanoma cDNA libraries to identify tumor-associated antigens that cure melanoma. Nat Biotechnol (2012) 30:337–43 10.1038/nbt.2157 22426030 PMC3891505

[125] Palucka K Banchereau J Dendritic-cell-based therapeutic cancer vaccines. Immunity (2013) 39:38–48 10.1016/j.immuni.2013.07.004 23890062 PMC3788678

[126] Taube JM Anders RA Young GD Xu H Sharma R McMiller TL Colocalization of inflammatory response with B7-h1 expression in human melanocytic lesions supports an adaptive resistance mechanism of immune escape. Sci Transl Med (2012) 4:127ra37 10.1126/scitranslmed.3003689 22461641 PMC3568523

[127] Lennerz V Fatho M Gentilini C Frye RA Lifke A Ferel D The response of autologous T cells to a human melanoma is dominated by mutated neoantigens. Proc Natl Acad Sci U S A (2005) 102:16013–8 10.1073/pnas.0500090102 16247014 PMC1266037

[128] Srivastava PK Old LJ Individually distinct transplantation antigens of chemically induced mouse tumors. Immunol Today (1988) 9:78–83 10.1016/0167-5699(88)91269-8 3076762

[129] Matsushita H Vesely MD Koboldt DC Rickert CG Uppaluri R Magrini VJ Cancer exome analysis reveals a T-cell-dependent mechanism of cancer immunoediting. Nature (2012) 482:400–4 10.1038/nature10755 22318521 PMC3874809

[130] Vogelstein B Papadopoulos N Velculescu VE Zhou S Diaz LA Jr. Kinzler KW Cancer genome landscapes. Science (2013) 339:1546–58 10.1126/science.1235122 23539594 PMC3749880

[131] Heemskerk B Kvistborg P Schumacher TN The cancer antigenome. EMBO J (2013) 32:194–203 10.1038/emboj.2012.333 23258224 PMC3553384

[132] Castle JC Kreiter S Diekmann J Lower M van de Roemer N de GJ Exploiting the mutanome for tumor vaccination. Cancer Res (2012) 72:1081–91 10.1158/0008-5472.CAN-11-3722 22237626

[133] Hodi FS O’Day SJ McDermott DF Weber RW Sosman JA Haanen JB Improved survival with ipilimumab in patients with metastatic melanoma. N Engl J Med (2010) 363:711–23 10.1056/NEJMoa1003466 20525992 PMC3549297

[134] Wolchok JD Hodi FS Weber JS Allison JP Urba WJ Robert C Development of ipilimumab: a novel immunotherapeutic approach for the treatment of advanced melanoma. Ann N Y Acad Sci (2013) 1291:1–13 10.1111/nyas.12180 23772560 PMC3910157

[135] van RN van Buuren MM Philips D Velds A Toebes M Heemskerk B Tumor exome analysis reveals neoantigen-specific T-cell reactivity in an ipilimumab-responsive melanoma. J Clin Oncol (2013) 31:e439–42 10.1200/JCO.2012.47.7521 24043743 PMC3836220

[136] Gao Y Whitaker-Dowling P Griffin JA Barmada MA Bergman I Recombinant vesicular stomatitis virus targeted to Her2/neu combined with anti-CTLA4 antibody eliminates implanted mammary tumors. Cancer Gene Ther (2009) 16:44–52 10.1038/cgt.2008.55 18654610

[137] Dias JD Hemminki O Diaconu I Hirvinen M Bonetti A Guse K Targeted cancer immunotherapy with oncolytic adenovirus coding for a fully human monoclonal antibody specific for CTLA-4. Gene Ther (2012) 19:988–98 10.1038/gt.2011.176 22071969

[138] Zamarin D Holmgaard RB Subudhi SK Park JS Mansour M Palese P Localized oncolytic virotherapy overcomes systemic tumor resistance to immune checkpoint blockade immunotherapy. Sci Transl Med (2014) 6:226ra32 10.1126/scitranslmed.3008095 24598590 PMC4106918

[139] Kim HS Kim-Schulze S Kim DW Kaufman HL Host lymphodepletion enhances the therapeutic activity of an oncolytic vaccinia virus expressing 4-1BB ligand. Cancer Res (2009) 69:8516–25 10.1158/0008-5472.CAN-09-2522 19843856

[140] John LB Howland LJ Flynn JK West AC Devaud C Duong CP Oncolytic virus and anti-4-1BB combination therapy elicits strong antitumor immunity against established cancer. Cancer Res (2012) 72:1651–60 10.1158/0008-5472.CAN-11-2788 22315352

[141] Topalian SL Hodi FS Brahmer JR Gettinger SN Smith DC McDermott DF Safety, Activity, and Immune Correlates of Anti-PD-1 Antibody in Cancer. N Engl J Med (2012) 366:2443–54 10.1056/NEJMoa1200690 22658127 PMC3544539

[142] Workenhe ST Mossman KL Oncolytic virotherapy and immunogenic cancer cell death: sharpening the sword for improved cancer treatment strategies. Mol Ther (2014) 22:251–6 10.1038/mt.2013.220 24048442 PMC3916036

[143] Guo ZS Liu Z Bartlett DL Oncolytic immunotherapy: dying the right way is a key to eliciting potent antitumor immunity. Front Oncol (2014) 4:74 10.3389/fonc.2014.00074 24782985 PMC3989763

[144] Wolchok JD Kluger H Callahan MK Postow MA Rizvi NA Lesokhin AM Nivolumab plus ipilimumab in advanced melanoma. N Engl J Med (2013) 369:122–33 10.1056/NEJMoa1302369 23724867 PMC5698004

